# Association between Work-Related Trauma Exposure and Posttraumatic Stress Symptoms among Child Welfare Workers in Japan: A Cross-Sectional Study

**DOI:** 10.3390/ijerph18073541

**Published:** 2021-03-29

**Authors:** Mayumi Kataoka, Daisuke Nishi

**Affiliations:** Department of Mental Health, Graduate School of Medicine, The University of Tokyo, Tokyo 1130033, Japan; d-nishi@m.u-tokyo.ac.jp

**Keywords:** post-traumatic stress disorder, traumatic event, cumulative trauma exposure, resilience, event list, child welfare

## Abstract

Child welfare workers often experience work-related traumatic events and may be at risk of post-traumatic stress disorder (PTSD), which can hinder early interventions for child abuse. This study examined the association between each single work-related traumatic event experienced by child welfare workers and the cumulative number of traumatic event types with PTSD symptoms. A checklist of traumatic events was used to investigate work-related traumatic events. The PTSD checklist for DSM-5 (PCL-5) was used to screen for PTSD symptoms. Two multivariate analyses were performed. A total of 140 workers were included in the analyses. In the first multivariate analysis, the event, “Witnessed a parent violently beating, hitting, kicking, or otherwise injuring a child or the other parent during work” (β = 11.96; 95% CI, 2.11–21.80; *p* < 0.05) and resilience (β = −0.60; 95% CI, −0.84 to −0.36; *p* < 0.01) were significantly associated with PTSD symptoms, as was resilience in the second multivariate analysis (β = −0.60; 95% CI, −0.84 to −0.36; *p* < 0.01). The association between the cumulative number of event types and PTSD symptoms was not significant, but it was stronger when the cumulative number was four or more. The findings suggest the importance of reducing child welfare worker exposure to traumatic events.

## 1. Introduction

Previous research has found that victims of child abuse are more likely than nonvictims to suffer from mental and chronic illnesses, behavioral problems, interpersonal problems, and decreased productivity, with effects that continue into adulthood [1]. The economic losses from child abuse are $120 billion per year in the United States [2], while the social costs (e.g., costs of child social welfare services, medical costs) are $16 billion in Japan [3]. Child abuse is associated with public health problems.

Early intervention is essential to prevent the negative influences of child abuse, and child guidance centers play an important role in early intervention in Japan. Sixty percent of the consultations at child guidance centers in Japan are related to inadequate child care, such as abuse and neglect [4], and it is thought that child guidance center workers have many opportunities to come into contact with parents who are perpetrators of abuse and children who are victims. Contact with parents who cannot afford to obtain childcare may lead to a high frequency of work-related traumatic events, such as verbal abuse and violence, leading to post-traumatic stress disorder (PTSD) in workers. Previous research has shown that as the level of PTSD among child welfare workers increases, the child’s risk becomes increasingly underestimated [5]. PTSD may reduce the staff’s ability to judge risk and may hinder early intervention for abuse. However, few previous studies have examined PTSD among child welfare workers.

To date, previous research has revealed that the intensity of experiences and the length of exposure are associated with PTSD in single traumatic events [6,7,8]. Furthermore, previous studies in civil populations affected by war have shown dose–effect relationships between the cumulative number of traumatic events experienced and PTSD symptoms, pointing to the need to examine the effects of cumulative traumatic experiences as well as single traumatic events for people in settings with the potential for many different types of experiences [9,10,11]. Therefore, the effect of work-related traumatic events on PTSD among child guidance center workers may also need to be assessed for the cumulative effects of traumatic events, and not only to determine which type of traumatic event has a stronger effect. However, to the best of our knowledge, very few studies have assessed the effects of work-related traumatic events on PTSD among child welfare workers, and no previous studies have evaluated the cumulative effects of traumatic events.

The purpose of this study was to examine the association between the single and the cumulative number of types of work-related trauma events experienced by child guidance center workers and PTSD symptoms in Japan.

## 2. Materials and Methods

### 2.1. Participants

**Figure 1:**
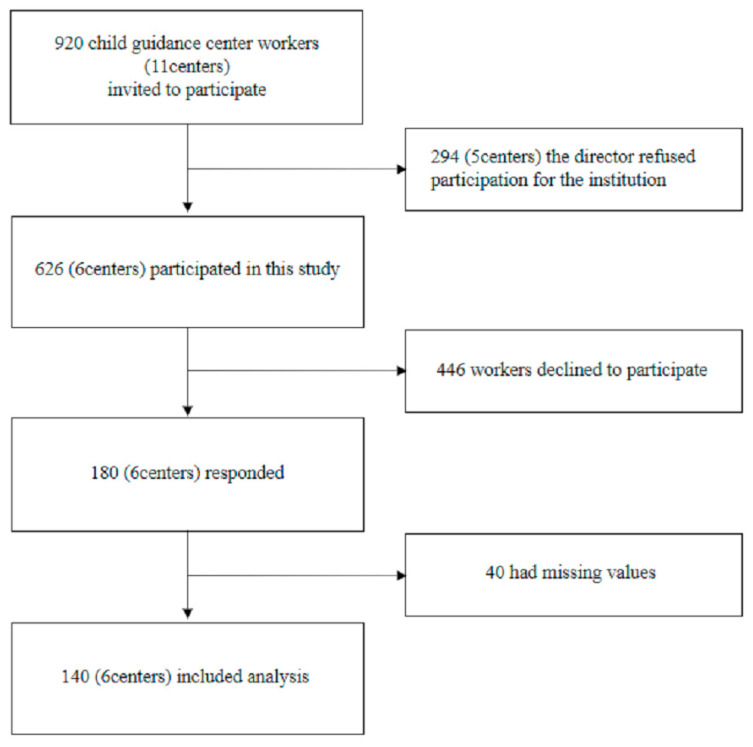
Flow diagram of the study.

A participant flow chart is shown in Figure 1. A total of 920 employees in 11 child guidance centers in a prefecture in Japan were invited to participate in this study. This prefecture is one of the most populous in Japan, and the number of abuse consultations was higher than the national average. The number of abuse cases handled by each worker was on par with the average of other prefectures with large populations. Two hundred and forty-nine employees in five centers declined to participate. Out of the 626 employees across six centers, 180 employees returned the questionnaires, and we excluded 40 because of missing values. Finally, 140 (response rate = 15.2%) were included in the analysis. Table 1 shows the characteristics of the participants.

### 2.2. Procedure

This study was a cross-sectional study using a self-administered questionnaire approved by the Ethics Committee of the University of Tokyo Graduate School of Medicine and School of Medicine (2019261NI).

A document requesting study cooperation was sent to each of the directors of 11 child guidance centers. Unless the director disagreed to participate in the study, we then sent documents about the purpose and the contents of this study, along with anonymous format questionnaires, to the director for the appropriate number of employees. After this, the directors distributed them to the employees. The document clearly stated that participation was nonmandatory and entirely voluntary, and stamped addressed envelopes were enclosed for the return of questionnaires to the researchers.

The inclusion criteria were (1) full-time and part-time adult employees working at the child guidance centers at the time of the survey and (2) employees who were physically and mentally able to consent. The exclusion criteria included employees who had been absent for longer than one month. These criteria were specified in the document about this study sent to the directors and employees.

Employees who agreed to participate in the study completed a questionnaire, checked the box to agree to participate in the study, and returned it to the researcher. Because respondents may have suffered from an intrusive re-experience after completing the questionnaire, for ethical considerations, all respondents were informed that referral to a hospital or clinic was available. A confirmation document about the questionnaire’s distribution was sent to the director before the return deadline for centers that had not returned questionnaires from their employees. The questionnaires were collected for one month, from February to March 2020.

### 2.3. Measures

#### 2.3.1. Outcomes: The PTSD Checklist for DSM-5 (PCL-5)

This self-administered questionnaire is widely used to screen for PTSD. The reliability and validity of the Japanese version have been confirmed in a Japanese population (Cronbach’s alpha = 0.97; r contrast-CV = 0.85; 95% CI, 0.847–0.862) [12]. It consists of 20 items about PTSD symptoms. Each item is scored on a five-point scale from 0 (not at all) to 4 (very). The total score takes a value from 0 to 80 points. Higher scores indicate a higher severity of symptoms of PTSD.

#### 2.3.2. Exposure: Work-Related Traumatic Events Checklist

To examine the experience of work-related traumatic events, we developed a novel checklist for child guidance center workers who had experienced traumatic events at work. This checklist was developed based on the findings of previous studies [13,14] and from interviewing child guidance center workers about their experiences of traumatic events encountered in their work. Only events that met the diagnostic criterion A for PTSD of the DSM-5 were selected [15] (p. 271). The criterion A for PTSD diagnostic criteria for DSM-5 was developed by the American Psychiatric Association. The items of diagnostic criterion A were the following: (1) directly experiencing the traumatic event(s); (2) witnessing, in person, the event(s) as it occurred to others; (3) learning that the traumatic event(s) occurred to a close family member or close friend; (4) experiencing repeated or extreme exposure to aversive details of the traumatic event(s). Finally, the checklist consisted of 14 traumatic events corresponding to the items of diagnostic criterion A: (1) directly experiencing the traumatic event(s) (e.g., “Victim of violence that could cause serious injuries, such as hitting, kicking, or being grabbed in the chest during work’”); (2) witnessing, in person, the event(s) as it occurred to others (e.g., “Witnessed colleagues being victims of verbal abuse or threats (throwing and breaking things) that felt dangerous or could cause serious injury or death during work”); (3) learning that the traumatic event(s) occurred to a close family member or close friend (e.g., “Learned that colleagues were stalked and locked themselves indoors for fear of the safety from the person involved in the work”); (4) experiencing repeated or extreme exposure to aversive details of the traumatic event(s) (e.g., “Repeatedly heard detailed stories about abuse during work”). The checklist format was adopted from the event checklist of the Clinician-Administered PTSD Scale for DSM-Ⅳ (CAPS).

The work-related traumatic events that participants described in the open-ended section were reviewed by two researchers and allocated to the type of events that were applied. Participants who had experienced a work-related traumatic event responded either once or two or more times per item. The cumulative number of types was estimated by assessing the number of different traumatic event types.

#### 2.3.3. Covariates

##### Brief Job Stress Questionnaire (BJSQ)

The Brief Job Stress Questionnaire (BJSQ) is a self-administered questionnaire widely used to evaluate job stressors. It is composed of questions about job demands (three items), job control (three items), social support from supervisors (three items), and social support from colleagues (three items). The reliability and validity of the Japanese version have been confirmed in a Japanese population (Cronbach’s alpha: job demands = 0.77, job control = 0.65, social support from supervisor = 0.79, and social support from colleague = 0.76) [16]. Each item is scored on a four-point scale from 1 (not at all) to 4 (very). The total score of each factor takes a value from 3 to 12 points. For job demands and job control, higher scores indicate more stress at work. For social support from supervisors and colleagues, higher scores indicate more support is available.

##### Tachikawa Resilience Scale (TRS)

The Tachikawa Resilience Scale (TRS) is a self-administered questionnaire developed to measure Japanese resilience. The reliability and validity of the Japanese version have been confirmed in a sample of Japanese company workers (Cronbach’s alpha = 0.82) [17]. We chose the TRS because this scale was developed as a concise measure of resilience, which is suitable for the Japanese, taking into account that qualities of resilience could be culturally sensitive. It consists of 10 items, each scored on a seven-point scale from 1 (strongly disagree) to 7 (strongly agree). The total score takes a value from 10 to 70 points. Higher scores indicate more resilience.

#### 2.3.4. Demographics and Characteristics

Demographics and characteristics such as age, years of work experience, and gender were measured. Previous studies have found these variables to be associated with PTSD [1,7,18,19]. In addition, we measured the qualifications held by participants because the risk of experiencing traumatic events in child guidance centers is likely to vary by qualifications.

### 2.4. Data Analysis

To examine the factors associated with PTSD symptoms and each variable, univariate analysis was performed. In the models for determining variables associated with the PTSD symptoms, two multivariate analyses were performed. We excluded the events 4 and 6 in Table 2 from the analyses because the events 1 and 4, and 4 and 5, were highly correlated, and the event 6 was a small prevalence (*n* = 4). Of the 14 traumatic events, 12 were used in the analyses. In the first multivariate analysis, each traumatic event experienced was transformed into a dummy variable (no experience) to examine the association between single work-related traumatic events experienced and PTSD symptoms. In the second multivariate analysis, the cumulative numbers of the types of traumatic events were transformed into dummy variables (no experience) to examine the cumulative effect of work-related traumatic events experienced on PTSD symptoms. The covariates gender (male) and qualifications (none) were also transformed into dummy variables. Missing values were not compensated. All analyses were performed using SPSS Statistics 26 (IBM, Tokyo, Japan).

## 3. Results

Table 1 shows the demographics and characteristics of the participants. The mean age and years of work experience were 43.98 (SD = 12.34) and 4.86 (SD = 4.17), respectively. Of the total number of participants, 25.7% were male and 74.3% were female, and 54.3% qualified for welfare. The mean PCL-5 and TRS scores were 10.75 (SD = 13.17) and 44.98 (SD = 10.01), respectively.

Table 2 and Table 3 show the percentages of each traumatic event experienced. The most experienced traumatic event was, “Repeatedly heard detailed stories about abuse during work” (70.0%), while 12.9% of the workers had no experience with work-related traumatic events, and 87.1% had at least one event.

Table 4 shows the results of the univariate and multivariate analyses. In the first multivariate analysis, the event, “During work, witnessed a parent violently beat, hit, kick, or otherwise injure a child or the other parent” (β = 11.96; 95%CI, 2.11–21.80; *p* < 0.05) and TRS (β = −0.60; 95%CI, −0.84 to −0.36; *p* < 0.01) were significantly associated with PCL-5 scores. In the second multivariate analysis, TRS (β = −0.60; 95%CI, −0.84 to −0.36; *p* < 0.01) was significantly associated with PCL-5 scores. The association between the cumulative number of event types and PCL-5 scores was not significant, but it was stronger when the cumulative number was four or more.

## 4. Discussion

This was the first study, to the best of our knowledge, to examine the association between PTSD symptoms and the single and the cumulative number of types of work-related trauma events experienced by child guidance center workers. The single traumatic event significantly associated with the severity of PTSD symptoms was, “During work, witnessed a parent violently beat, hit, kick, or otherwise injure a child or the other parent”.

A previous study of child welfare workers indicated that indirect traumatic events (working with children in distressing circumstances and being unable to do enough for the client, both specifically and generally) were more highly associated with trauma effects than were direct events (spoken abuse by a client to a worker and being placed in fear of personal safety by a client) [20]. This suggests that indirect events may be more toxic because they reflect workers’ lack of control and inability to impact clients’ lives adequately. A possible explanation is that these are assumed strength differences of peri-traumatic emotional responses noted as risk factors for PTSD [19] between indirect events and direct events. The event, “During work, witnessed a parent violently beat, hit, kick, or otherwise injure a child or the other parent”, seems an indirect event and made workers feel that their capacity to prevent abuse was ineffective, which might lead to workers’ feelings of inability.

In addition, such violence also seems to be a direct event. The event is likely to occur in a closed space where the consultation takes place, such as a consultation room or the client’s home. Witnessing the violence in a closed space might lead the worker to fear being caught up in that violence. These are also assumed peritraumatic emotional responses. Thus, this event might create more stronger peritraumatic emotional responses than other events.

However, the estimated value had a wide 95% confidence interval range. This may be due to the small number of workers who had experienced this event (*n* = 8). Further research of a longitudinal design with random sampling and a large number of participants will be needed to validate this result.

Although the association between the cumulative number of event types and the severity of PTSD symptoms was not significant, it was stronger when the cumulative number was four or more. This is partly consistent with previous studies’ findings, which showed dose–effect relationships between the cumulative number of types of traumatic events experienced and lifetime PTSD symptoms [9,10]. A similar relationship pattern was also seen in child guidance center workers.

In professions that may experience many types of work-related traumatic events, such as child guidance center work, we may need to pay attention to the cumulative number of events and not just single events. Further research among the occupations likely to experience various types of work-related traumatic events will be needed to validate this result.

It may be important to reduce the experience of witnessing violence or other types of traumatic events in child guidance center workers to avoid having the negative effects of PTSD symptoms affect their performance. A previous study of child welfare workers suggested that consideration of the causes and effects of violence, reflecting support for planning and a commitment of agencies to worker safety, is essential to prevent violence of clients [21]. A study of healthcare workers in emergency departments found that training (focused on constructing a relationship with the patient, improving the worker’s communication skills, and accurately reporting violent incidents) and implementing workplace design effective in minimizing stressful conditions of patients should be prioritized to prevent and manage patient violence [22]. Further research may be needed to assess the commitment and measures of child guidance centers to prevent work-related traumatic events and to determine how effectively they work.

Resilience had a significant negative association with PTSD symptoms. This is consistent with previous studies showing resilience to be a protective factor against PTSD, especially among the occupations that are likely to experience work-related traumatic events (e.g., firefighters, police officers, and intensive care unit nurses) [23,24,25,26,27]. On the contrary, another previous study suggested that there is no ultimate resilience to traumatic stress, and that the repeated occurrence of traumatic stress has a cumulative damaging effect on the mental health of the victim, because all develop PTSD once the trauma load reaches a certain threshold [10]. Therefore, to prevent PTSD in child guidance center workers who may experience various types of traumatic events, it may be essential to reduce even one traumatic event.

These results were obtained from workers in six centers in one prefecture, which is one of the most populous, and the number of abuse consultations was higher than the national average in Japan. Although the number of abuse cases handled by each worker was on par with other prefectures with a large population, the number of workers per center was greater than that of other prefectures. For these reasons, participants might be more likely to experience traumatic events, especially indirect events, such as witnessing and learning their colleagues’ traumatic events, compared to other prefectures. Thus, workers’ characterization of traumatic events may differ depending on the prefecture and workplace. To confirm this study’s results, further research is needed to investigate the differences in traumatic events experienced by workers in each prefecture and workplace and how they affect PTSD symptoms.

This study has some limitations. First, the response rate was low (15.2%), and the sample size was modest (*n* = 140). There was the possibility of selection bias. Participants may have been biased toward those interested in their mental health. As a result, the association between traumatic events and PTSD symptoms might be overestimated. Second, generalization was limited because the results were obtained from workers in six centers in one prefecture in Japan, due to the possibility of the difference in workers’ traumatic event experiences in each prefecture. Third, there were other confounding factors for PTSD symptoms for which we did not collect information from participants (e.g., history of psychiatric illness and peritraumatic factors). Failure to adjust for these confounding factors might have influenced the results. Fourth, PTSD symptoms might be associated with other lifetime traumatic events rather than work-related events. In this case, the effect of work-related traumatic events on PTSD symptoms might be overestimated. Fifth, the work-related traumatic event checklist was created based on interviews with child guidance center staff and previous studies, but there is a possibility that the checklist might not be comprehensive and might not cover some work-related traumatic events. Therefore, there might be traumatic events for which the impacts on PTSD symptoms were not assessed in this study. Sixth, because this study was a cross-sectional study, it was impossible to determine a causal relationship.

## 5. Conclusions

The witnessing of violence between clients by workers was significantly associated with the severity of PTSD symptoms. The association between the cumulative number of event types and PTSD symptoms was not significant, but it was stronger when the cumulative number was four or more. Resilience was found to be a protective factor against PTSD symptoms. It may be important to reduce the experience of witnessing violence or other types of traumatic events in child guidance center workers to avoid having the negative effects of PTSD symptoms affect their performance.

## Figures and Tables

**Table 1:** Demographics and characteristics of the participants in this study.

Variables	*n*	%	Mean	SD
Age			43.98	12.34
Years of work experience			4.86	4.17
Gender				
Male	36	25.7		
Female	104	74.3		
Qualification				
None	9	6.4		
Welfare ^a^	76	54.3		
Psychology	21	15.0		
Medical	5	3.6		
Others	12	8.6		
Multiple	17	12.1		
Job stressors				
Job demand			10.27	1.94
Job control			7.25	2.10
Social support from supervisors			7.79	2.05
Social support from colleagues			8.36	1.96
TRS score			44.98	10.01
PCL-5 score			10.75	13.17

Notes. *n* = 140. TRS, Tachikawa Resilience Scale; PCL-5, the PTSD Checklist for the DSM-5. ^a^ The qualification of worker who provide consultation on child abuse in Japan.

**Table 2:** Number and percentage of workers who experienced work-related traumatic events.

	Once (a)		Two or More Times (b)		(a) + (b)	
	*n*	%	*n*	%	*n*	%
Directly experiencing the traumatic event(s)						
1. Victim of violence that could cause serious injuries, such as hitting, kicking, or being grabbed in the chest during work	11	7.9	15	10.7	26	18.6
2. Victim of verbal abuse or threats (throwing and breaking things) that felt dangerous or could cause serious injury or death during work	10	7.1	22	15.7	32	22.8
3. Stalked and locked indoors for fear of safety from the person involved in the work	4	2.9	1	0.7	5	3.6
Witnessing, in person, the event(s) as it occurred to others						
4. Witnessed colleagues being victims of violence that could cause serious injuries, such as hitting, kicking, or being grabbed in the chest during work	6	4.3	18	12.9	24	17.2
5. Witnessed colleagues being victims of verbal abuse or threats (throwing and breaking things) that felt dangerous or could cause serious injury or death during work	4	2.9	13	9.3	17	12.2
6. Witnessed colleagues stalked and locked indoors for fear of safety from the person involved in the work	2	1.4	2	1.4	4	2.8
7. Witnessed a parent violently beat, hit, kick, or otherwise injure a child or the other parent during work	3	2.1	5	3.6	8	5.7
8. Witnessed a parent verbally abusing or threatening that felt dangerous or could cause serious injury or death to a child or the other parent during work	5	3.6	14	10.0	19	13.6
Learning that the traumatic event(s) occurred to a close family member or close friend						
9. Learned that colleagues were victims of violence that could cause serious injuries, such as hitting, kicking, or being grabbed in the chest during work	10	7.1	20	14.3	30	21.4
10. Learned that colleagues were victims of verbal abuse or threats (throwing and breaking things) that felt dangerous or could cause serious injury or death during work	15	10.7	21	15.0	36	25.7
11. Learned that colleagues were stalked and locked indoors for fear of the safety from the person involved in the work	4	2.9	5	3.6	9	6.5
Experiencing repeated or extreme exposure to aversive details of the traumatic event(s)						
12. Repeatedly heard detailed stories about abuse during work	5	3.6	93	66.4	98	70.0
13. Heard that a child or parent died who had been involved in work	22	15.7	51	36.4	73	52.1
14. Contact with children repeatedly being protected from abuse or severe injury due to abuse, or who were too thin, causing suspicion of a danger to life or malnourishment during work	8	5.7	66	47.1	74	52.8

Note. *n* = 140.

**Table 3:** Cumulative number of types and percentages of work-related traumatic events experienced.

Cumulative Number of Types of Work-Related Trauma Events Experienced ^a^	*n*	%
0	18	12.9
1	23	16.4
2	16	11.4
3	34	24.3
4	17	12.1
5	13	9.3
≥6	19	13.6

Note. *n* = 140. ^a^ The experiences of the events 4 and 6 were excluded from the number because the events 1 and 4, and 4 and 5, were highly correlated, and the event 6 was a small prevalence. They were excluded from the analyses.

**Table 4:** Results of univariate and multivariate analyses.

	Univariate			Multivariate (First)			Multivariate (Second)		
	Beta	SE	95% CI	Beta	SE	95% CI	Beta	SE	95% CI
Age	−0.20	0.08	(−0.38, −0.02) *	−0.03	0.10	(−0.23, 0.16)	−0.02	0.09	(−0.21, 0.17)
Years of work experience	−0.36	0.26	(−0.89, 0.16)	−0.30	0.30	(−0.91, 0.29)	−0.35	0.29	(−0.93, 0.21)
Gender, women	−0.52	2.55	(−5.57, 4.53)	−0.88	2.47	(−5.77, 4.01)	−1.74	2.43	(−6.55, 3.07)
TRS score	−0.59	0.10	(−0.79, −0.39) **	−0.60	0.12	(−0.84, −0.36) **	−0.60	0.11	(−0.84, −0.36) **
Qualification									
None	Reference			Reference			Reference		
Welfare ^a^	7.00	4.59	(−2.08, 16.09)	4.37	4.48	(−4.50, 13.25)	5.82	4.38	(−2.86, 14.50)
Psychology	4.01	5.19	(−6.25, 14.28)	5.78	5.02	(−4.17, 15.74)	6.23	4.93	(−3.54, 16.01)
Medical	−2.88	7.27	(−17.27, 11.49)	1.82	7.10	(−12.23, 15.89)	3.46	7.07	(−10.54, 17.48)
Others	−1.38	5.74	(−12.75, 9.98)	2.41	5.48	(−8.45, 13.29)	4.08	5.37	(−6.55, 14.73)
Multiple	5.58	5.37	(−5.04, 16.21)	7.40	5.18	(−2.87, 17.68)	7.65	5.13	(−2.50, 17.82)
Job stressors									
Job demand	2.04	0.55	(0.95, 3.13) **	1.09	0.65	(−0.20, 2.40)	1.15	0.65	(−0.14, 2.44)
Job control	−0.95	0.52	(−1.99, 0.08)	0.04	0.61	(−1.17, 1.25)	0.04	0.58	(−1.11, 1.21)
Social support from supervisors	−0.19	0.54	(−1.27, 0.88)	0.83	0.68	(−0.52, 2.19)	0.68	0.67	(−0.64, 2.00)
Social support from colleagues	−0.85	0.56	(−1.97, 0.27)	−0.30	0.69	(−1.67, 1.06)	−0.25	0.68	(−1.61, 1.10)
Type of traumatic event experienced ^b^									
0	Reference			Reference					
1	−2.84	3.66	(−10.09, 4.41)	−0.43	3.35	(−7.07, 6.21)			
2	0.51	3.22	(−5.86, 6.89)	2.52	2.91	(−3.24, 8.30)			
3	−3.98	6.32	(−16.49, 8.52)	−8.31	5.75	(−19.70, 3.07)			
5	3.88	4.30	(−4.62, 12.40)	1.53	3.87	(−6.15, 9.21)			
7	12.29	5.43	(1.53, 23.06) *	11.96	4.97	(2.11, 21.80) *			
8	−3.30	3.90	(−11.02, 4.42)	−1.65	3.52	(−8.63, 5.31)			
9	0.75	3.23	(−5.63, 7.15)	1.74	2.94	(−4.09, 7.58)			
10	−1.17	2.82	(−6.76, 4.41)	−1.31	2.56	(−6.40, 3.76)			
11	5.52	5.04	(−4.45, 15.51)	4.46	4.55	(−4.56, 13.49)			
12	0.93	2.75	(−4.50, 6.37)	0.27	2.68	(−5.04, 5.59)			
13	−0.13	2.49	(−5.07, 4.81)	−0.84	2.42	(−5.64, 3.96)			
14	1.53	2.52	(−3.47, 6.53)	1.84	2.36	(−2.83, 6.52)			
Cumulative number of types of traumatic events experienced									
0	Reference						Reference		
1	1.93	4.14	(−6.26, 10.13)				3.22	3.72	(−4.14, 10.59)
2	0.54	4.52	(−8.40, 9.50)				−1.55	4.15	(−9.77, 6.66)
3	0.19	3.84	(−7.39, 7.79)				0.40	3.70	(−6.93, 7.74)
4	−0.30	4.45	(−9.11, 8.51)				2.04	4.42	(−6.71, 10.81)
5	4.11	4.79	(−5.37, 13.59)				6.51	4.64	(−2.68, 15.70)
≥6	8.00	4.33	(−0.56, 16.57)				8.24	4.20	(−0.07, 16.56)
*R* ^2^				0.36 **			0.34 **		
Adjusted *R*^2^				0.23 **			0.23 **		

Note. *n* = 140. Dependent variable=PTSD symptoms. The experiences of the events 4 and 6 in Table 2 were excluded from the analyses because the events 1 and 4, and 4 and 5, were highly correlated, and the event 6 was a small prevalence. CI, confidential interval. SE, standard error. ^a^ The qualification of worker who provide consultation on child abuse in Japan. ^b^ The contents of the number of types of traumatic events experienced correspond to the contents of Table 2. * *p* < 0.05; ** *p* < 0.01.

## Data Availability

Data sharing is not applicable to this article. The ethics committee approved the study provided that the data were used only by the authors.
